# Scoping Pleiotropy of CK2 in Musculoskeletal Disorders for a Novel Targeting Approach

**DOI:** 10.3390/kinasesphosphatases2010004

**Published:** 2024-01-31

**Authors:** Venu Pandit, Kailey DeGeorge, Anja Nohe

**Affiliations:** 1 Department of Biological Sciences, University of Delaware, Newark, DE 19716, USA

**Keywords:** Casein Kinase II, Musculoskeletal, Osteoarthritis, Osteoporosis, CX-4945, CIBG-300, Cell Signaling, Drug Development, Targeted therapy

## Abstract

Protein kinase CK2 (CK2) influences one-fifth of the cellular phosphoproteome. It regulates almost all cellular pathways and thus is a critical switch between biological processes in a cell. Inhibition of CK2 reverses oncogene addiction of tumor and alters tumor microenvironment. The success of this strategy and its clinical translation opens new opportunities. Targeting CK2 in musculoskeletal disorders is promising. Clinical manifestations of these disorders include dysfunctional inflammation, dysregulated cell differentiation, and senescence. Processes regulated by CK2 include all of these. Its emerging role in senescence also indicates its function's centrality in cellular metabolism. This review summarizes considerations for targeting CK2 in musculoskeletal disorders. We have discussed the implications of CK2-regulated processes in musculoskeletal disorders.

## 1. Introduction

Musculoskeletal disorders affect skeletal muscle, bone, cartilage, and connective tissue. Disorders such as Osteoporosis (OP), Osteoarthritis (OA), and Rheumatoid arthritis (RA) can cause chronic pain, hamper mobility, and reduce quality of life. They are also among the costliest co-morbidities^1^. Traditionally, musculoskeletal disorders were identified as structural dysfunctions of respective physiologies. However, recently, metabolic dysregulation has come up as a significant risk factor in these diseases. Metabolic dysregulation is caused by the defective regulation of biochemical pathways required for homeostasis^2; 3^. Emerging studies are investigating the underlying mechanism by which metabolic disorders increase the severity of musculoskeletal disorders. Rheumatoid arthritis (RA) is an autoimmune disorder affecting joints. Researchers are exploring the link between autoimmunity and altered metabolism^4; 5^. Likewise, Osteoarthritis (OA) is characterized by progressive degeneration of joints. The pathogenesis of OA is affected by metabolic disorders^6; 7^. Small molecular metabolites are assessed for OA-associated biomarker identification^8^. Altered lipid metabolism and immune response during obesity increase the severity of OA symptoms^9^. Osteoporosis (OP) is a disorder associated with low bone mineral density. Several studies investigate the complex relationship between bone homeostasis and metabolic disorders, especially in the case of OP^10; 11; 12; 13; 14; 15^. Patients diagnosed with OP are at a higher risk of fractures. An impaired immune system affects the natural process of fracture healing^16^. Defects in fracture healing in obese and insulin-resistant individuals are attributed to the dysregulated innate immunity during healing-associated inflammatory response^17^.

Protein kinase CK2 (formerly called “Casein Kinase 2”) is a ubiquitous serine/threonine kinase. The kinase has no extracellular activator^18^. It is not regulated hierarchically by other kinases of vertical signaling cascades. Thus, the regulatory role of CK2 is not limited to the activation of molecular signaling from the plasma membrane to the nucleus. Instead, it crosswise associates and integrates multiple pathways^19; 20^. Hence, it is a ‘Lateral player’ in many biochemical pathways ^21; 22^. Expression and activity of CK2 are dysregulated in musculoskeletal disorders^23^. Deviation in metabolic processes affects tissue homeostasis and response to endocrine regulation^24^. The role of the inflammatory system and senescence in these diseases is also linked with metabolic dysregulation^25; 26^. Moreover, the role of CK2 in modulating immune response, adipogenesis, and mitochondrial function has long been studied^27; 28; 29^. The kinase can identify and phosphorylate a vast range of substrates, estimated to be more than 400 today^30;31^. It is predicted to account for almost 20% of the phosphoproteome of cells^32^. Since CK2 is a lateral player, the effects of its dysregulation are manifold.

CK2 is ubiquitously present and expressed in almost all types of cells. The mechanism of pathway regulation by CK2 is different from that of other kinases. The following factors summarize the complexity of CK2’s function. First, while most kinases are activated in response to particular stimuli, CK2 is constitutively active ^33^ and requires no specific stimulus^18; 34; 35^. Second, the localization and form of CK2 changes based on cell type and metabolic status^36^. CK2 has two catalytic subunits, CK2α and CK2α’, and a regulatory CK2β subunit. *In vivo*, it exists as a heterotetramer^37^. The tetramer form of CK2 is also termed CK2 holoenzyme. Holoenzyme comprises catalytic subunits α and α’ and two regulatory β subunits. The kinetics of holoenzyme formation and cellular distribution of these subunits change under different stimuli^38^. Third, the β subunit is not strictly a regulatory subunit. It is required to recognize substrates and structural stability of catalytic subunits. However, few substrates are phosphorylated without the β subunit, which makes its role elusive. Fourth, the catalytic subunits CK2α and CK2α’ are almost the same in structure, but their involvement in catalytic activity is different^39^. One is preferred over the other in some processes. Finally, the expression of subunits is regulated by various growth factors, and a single subuniťs expression level affects the expression and activity of other subunits.

The only clinically approved small molecular inhibitor of CK2, Silmitasertib (CX-4945), is a first-in-class small molecule of its kind^40^. It is an ATP-competitive inhibitor having the highest level of selectivity for CK2. Its use in cancer therapies is under investigation. However, like most currently available CK2 inhibitors, it inhibits CK2 catalytic activity completely. Controlling the inhibition of CK2 activity during specific cellular processes while sparing other activity is not possible. Furthermore, complete inhibition of the catalytic activity of CK2 causes cytotoxicity. This is due to the cells' dependence on the basal phosphorylation level of CK2 substrates for survival^35^. Cytotoxicity is not always desirable, especially in treating musculoskeletal disorders. The technology of modulating the phosphorylation of CK2 substrates taking part in disease pathogenesis rather than inhibiting overall kinase activity is emerging. Focusing on specific CK2-substrate interaction will help bring a pathology-tailored approach for musculoskeletal disorders^41^.

One such peptide inhibitor of CK2 named CIGB300 inhibits phosphorylation of specific CK2 substrates. This synthetic peptide binds to the phosphoacceptor domain of CK2^42^. It was first discovered as an effective peptide in treating cervical cancer. It is also effective in treating Large Cell Lung Carcinoma (LCLC), Non-Small Cell Lung Cancer (NSCLC), advanced cervical cancer, and Acute Myeloid Leukemia^43; 44; 45; 46^. Due to its substrate-specific inhibition, it is highly effective and has mild side effects. This drug is under investigation in Phase 2 clinical trials, and studies about its effectiveness will soon be available^29^. The substrate-specific rather than the global inhibitory effect of CIGB300 is thus emerging in cancer treatment.

Disease-modifying drugs help reverse the symptoms of disorders; they do so because they are designed to target molecular interactions critical for pathogenesis^47^. This approach re-directs metabolic pathways towards homeostasis. Few disease-modifying drugs like Sprifermin (recombinant human FGF18, rhFGF18) for OA and methotrexate for RA are available to treat musculoskeletal diseases^48; 49^. Interventions that can restore dysregulated metabolism in these diseases are necessary ^47^. CK2 is a lateral player, restoring its activity will effectively re-direct the metabolism to a healthy state. However, Targeting CK2 is challenging due to the complexity of its function^21; 22; 50^. Efforts to reveal the complexities of CK2 function in musculoskeletal disorders will help design disease-modifying therapeutics.

For the development of new drugs, the success rate of candidate molecules to move from bench to bedside is only 10%, despite the robustness of preclinical studies and perfect administration of clinical trials. Moreover, an excellent pre-clinical performer may be withdrawn due to the long processing time between drug discovery and commercial production. Substantial costs are involved with this process, and more importantly, the high failure rate delays the advances in better treatment for affected individuals. Lack of biomarker identification, off-target activity, and excess toxicity are a few of the challenges identified that can be addressed while designing new candidates. This process can utilize advanced knowledge of the molecular interactions between the drug and target^51^. In the case of CK2, many small molecular inhibitors have been discovered, most of which are investigated for the treatment of cancers. Yet, very few are approved for clinical use, partly due to their high cytotoxicity. Re-purposing the knowledge about these CK2 inhibitory molecules for treating musculoskeletal disorders is possible. Understanding the critical interactions of CK2 with its substrates in musculoskeletal diseases becomes essential.

In this review, first, we discuss the implications of CK2 in musculoskeletal disorders. Next, we discuss mechanisms of target identification by CK2 and the distinct functions of its subunits. Then, we describe currently available inhibitors of CK2 and their mechanism of action. Finally, we discuss a novel disease-modifying approach for targeting CK2-substrate interactions. This approach achieves CK2 activity modulation using biomimetic peptides targeting specific interactions of CK2 within the signal transduction cascade.

## 2. Implication of CK2 in Musculoskeletal disorders

### 2.1. Rheumatoid arthritis –

Rheumatoid arthritis (RA) is an autoimmune disorder affecting joints. The exact mechanisms through which RA develops have yet to be fully understood^52; 53^. One leading hypothesis is that the interactions between epigenetic modifications and environmental influences facilitate self-antigens production and induce autoimmunity. Self-antigens are then modified by citrullination^5^. As the body cannot recognize citrullinated antigens, the modified self-antigens are then recognized by antigen-presenting cells, which transport them to the lymph nodes. In the lymph nodes, helper T cells and B cells are activated through co-stimulation. The B cells differentiate into plasma cells that produce antibodies against the self-antigens^54^. In this manner, the body produces antibodies against self-antigens and induces tissue inflammation^55^. Synovial hyperplasia and similar disorders may also release cytokines contributing to joint inflammation and autoimmunity. Specifically, the synovial fluid and joint capsule become inflamed during RA, contributing to bone and cartilage erosion ^56^. During RA, the balance between the Type I T Helper (Th1) and Type 2 T Helper (Th2) cells is disrupted in favor of the Th1 cells, causing inflammation. Type 17 T Helper (Th17) cells induce the proliferation of fibroblast-like synoviocytes (FLS)^57^. FLSs contribute to the progression of RA. FLSs are found in the synovial intimal lining of healthy tissue; they secrete products that contribute to the synovial fluid and articular cartilage. In RA, they secrete cytokines that promote inflammation and proteases that degrade cartilage ^58^.

Protein kinase CK2 plays a role in RA through CK2α’s control of the Interleukin-12 (IL-12) receptor, which regulates the IL-STAT4 signaling pathway through which Th1 cell differentiation is promoted. The IL-STAT4 pathway is regulated primarily by the expression of IL-12R in CD4+ T cells, though it is also regulated, to a lesser extent, by interferon-α (IFN-α) and interleukin 23 (IL-23). The phosphorylation of the STAT4 pathway induces downstream signaling that ultimately facilitates Th1 cell differentiation ^59^. CK2α, CK2α’, and CK2β promote Th17 cell differentiation. CK2α also regulates Th1 cell differentiation and suppresses the activity of Th2 cells ^59; 60^.

### 2.2. Osteoarthritis

Osteoarthritis (OA) is a degenerative disease of the articular cartilage, which is increasingly common with advanced age, especially in women. OA is associated with loss in the number and activity of chondrocytes through increased apoptosis. Reduced activity of chondrocytes reduces the repair of collagen matrix and diminishes the structural integrity of the cartilage. Oxidative stress is another hallmark of OA. Metabolic disorders affect the severity of OA. Increased serum glucose levels due to type 2 diabetes and metabolic syndrome increase advanced glycation products (AGESs) levels. The AGES are associated with the collagen matrix of cartilage and cause structural damage. They also interact with receptors of AGES called RAGE and cause oxidative stress in chondrocytes^61^. Excess reactive oxygen species (ROS) production leads to oxidative stress response, increasing chondrocyte senescence^62^.

CK2 is an inhibitor of apoptosis, and its expression is reduced in chondrocytes of patients diagnosed with OA. Inhibition of CK2, sensitized chondrocytes to Tumor Necrosis Factor-alpha (TNFα) - mediated cell death^63^. Parathyroid hormone-related protein (PTHrP) is a Parathyroid hormone homolog. It protects chondrocytes from undergoing excessive apoptosis ^64^. PTHrP exerts its protective activity against mitochondria-dependent apoptotic pathway via upregulation of CK2 activity^65;66^. PTHrP is localized to nuclei due to its mid-region bipartite nuclear targeting sequence (NTS)^67^. In one study, treatment with synthetic peptide PTHrP-NTS (the NTS region of PTHrP) protected HEK293 cells from TNFα activated apoptosis. Exogenous NTS increased nuclear accumulation of CK2. Nuclear retention and activity of CK2 were enhanced. Similar results were obtained in cells treated with intact PTHrP ^68^. CK2 is also involved in the oxidative stress response. CK2 signaling is essential for activating transcription factor NF-E2-related factor 2 (Nrf2). The Nrf 2 signaling is crucial for redox balancing within cell ^69^. It reduces cellular ROS levels and suppresses nuclear factor-κB (NF-κB) localization in the nucleus. Nrf2 upregulates expression of Heme oxygenase-1 (HO-1). Enzyme HO-1 is vital in heme degradation and countering oxidative stress response ^70^. This enzyme gets activated in chondrocytes in response to peroxynitrite-induced oxidative stress ^71^. In another study, inhibition of the catalytic activity of CK2 with 4,5,6,7-terabromo-2-azabenzimidazole (TBB) and 5,6-dichlorobenzimidazole 1-β-D-ribofuranoside (DRB) induced senescence and apoptosis in chondrocytes. Overexpression of HO-1 reduced the TBB-induced senescence. Chondrocytes overexpressing HO-1 had reduced sensitivity towards TBB-induced senescence. The knockdown of CK2 reduced type II collagen and increased β catenin expression ^72^. The small heat shock protein αB-crystallin is a mediator for the expression of chondrogenic Bone Morphogenetic Protein-2 and collagen type II. Its expression is downregulated in OA^73^. Chondrocytes treated with CK2 inhibitors and αB-crystallin siRNA were sensitized to apoptosis. However, αB-crystallin had a protective effect. Its expression was modulated, and cellular localization was changed after CK2 inhibition ^74^. The role of αB-crystallin in aiding CK2 to prevent chondrocyte apoptosis should be explored further. Thus, evidence suggests that CK2 protects chondrocytes from oxidative stress and apoptosis.

### 2.3. Bone fracture

Healthy bone is excellent at self-healing in the event of injury. The process occurs roughly through the following phases. The first is the inflammatory phase and hematoma formation. Physical rupture of the bone causes infiltration of immunoregulatory cells, such as Macrophages, at the site. These cells secrete inflammatory cytokines IL-1, IL-6, and TNFα during the early phases of healing^75^. These factors recruit mesenchymal stem Cells (MSC) to the fracture site. MSCs undergo chondrogenic and osteogenic differentiation through secreted growth factors. Next, angiogenesis and soft callus formation begin, at which point the central region of the fracture undergoes endochondral ossification during this phase^76^. The MSCs undergo differentiation into Osteo-chondroprogenitor cells^77^. Thus, formed chondrocytes form the cartilaginous matrix. Angiogenesis and calcification of the cartilaginous matrix by hypertrophic chondrocytes marks the start of the next phase of hard bone formation. In this phase, the cartilage gets replaced by bone by hard calcification. Here, the activity of osteoblasts increases. Finally, in the last phase, bone remodeling occurs. Here, the coordinated function between bone matrix-secreting osteoblasts and bone-resorbing osteoclasts takes place ^64^. Bone remodeling is essential in maintaining bone shape. During aging, Macrophages, chondrocytes, and osteocytes secrete senescence-associated factors^78^. Their secretion prolongs the inflammatory phase and hampers the bone healing process ^79; 80^.

The bone morphogenic protein (BMP) pathway is essential for fracture healing ^81 82^. BMP ligands are critical growth factors for bone, cartilage, joint development, and homeostasis. They drive MSC differentiation. BMP-2 stimulation of senescent bone marrow mesenchymal stem (BMSC) cells activated adipogenic and cell death pathways like NF-kB or p38-mitogen activated protein kinase (MAPK). In non-senescent bone marrow mesenchymal stem cells (BMSCs), BMP-2 activated bone-forming pathways such as SMAD, BMP, and TGFβ ^83^. CK2 negatively regulates BMP-2-activated signaling ^84^.

Casein kinase 2 interacting protein-1 (CKIP-1) is a negative regulator of BMP signaling. ^85^. CKIP-1 interacts with the CK2α subunit and regulates signaling between CK2α and effector molecules of BMP signaling ^86;87^. CKIP-1 is known to inhibit BMP signaling by promoting Smad1 ubiquitination. The suppression of BMP signaling leads to suppressed osteogenic differentiation ^23^. The CKIP-1 knockout mice had abnormally high levels of bone mass. The effects of CKIP-1 on bone growth and repair were age-dependent, with CKIP-1 having a more substantial impact on the bone mass of 18-month-old mice and comparatively little effect on 2-month-old mice. CKIP-1 is a negative regulator of osteogenic differentiation, and age-related inflammation causes an upregulation of CKIP-1, although the exact mechanisms of this upregulation remain unclear ^88; 89^. Inhibition of interaction between CK2α and CKIP-1 is a possible therapeutic strategy for bone fractures.

### 2.4. Osteoporosis

Osteoporosis (OP) is a degenerative disease that, as of 2023, affects more than 200 million people. The incidence rate of OP is 1.7% of men and 26% of women over age 50 worldwide ^65^. OP is associated with a loss in bone mass and mineral density. This weakens the bones and predisposes them to fractures. It is a musculoskeletal disorder that is very closely related to metabolic dysregulation. OP is characterized by a disruption in the balance between osteoblasts and osteoclasts, cells that generate bone and cells that degrade bone, respectively. One common type of OP is glucocorticoid-induced osteoporosis (GIO). GIO is diagnosed in patients undergoing treatment with glucocorticoids for inflammatory or auto-immune diseases. Glucocorticoids inhibit osteoblast function and decrease the vascularity of the bone ^66^. In one study, primary cells from human patients and mice with GIO had elevated levels of CKIP-1 and reduced levels of Smad1/5 compared to controls. CKIP-1 is a non-enzymatic protein that regulates the CK2α subunit of protein kinase CK2. It can be utilized in the localization of protein kinase CK2 in cell ^67^. *In vitro*, elevated expression and activity of CKIP-1 in osteoblasts inhibited Smad-dependent BMP signaling^23^. CKIP-1 regulates BMP signaling and can recruit CK2α to the plasma membrane. Hence, its overexpression can affect the activation of BMP signaling in osteoblasts and osteoclasts. CKIP-1’s suppression of BMP signaling inhibits and affects the differentiation of MSC into osteoblasts. These alterations contribute to the reduction in bone mineralization observed in GIO patients. Suppressing the interaction between CKIP-1 and interaction with CK2 could be a novel therapeutic strategy for treating GIO. Upregulation of BMP signaling can increase bone formation and repair ^16^. Blocking of the interaction between CK2 and CKIP-1 may induce BMP signaling^68^. A challenge in creating osteoporosis therapeutics is maintaining the optimal balance between osteoblasts and osteoclasts, cells that generate and degrade bone, respectively. Ideally, a therapeutic for OP would increase osteoblast activity while decreasing osteoclast activity, inducing bone formation and repair.

## 3. Inhibition of CK2

### 3.1. Molecular function of CK2 –

#### 3.1.1. Structure of CK2 and substrate recognition

CK2 was discovered as a mitochondrial kinase in 1954; since then, its function has been studied in various biological processes^90^. Recent research has identified its new substrates and allowed us to expand our knowledge about its regulation of biological processes.

The catalytic subunits of CK2 are acidophilic. Its substrates often have acidic amino acids near the phosphorylation site. The consensus sequence on the phosphorylation target ([pS/pT]-(P)-x-[E/D] or [pS/pT]-(P)-x-pS is identified as the minimum identification sequence required for target identification ^30; 91^. However, due to the short length of this sequence and flexibility for amino acids in positions second and third, the frequency of occurrence of this sequence across all proteins is high. With the consensus prediction software PrositePlus, the number of eukaryotic targets comes out to be 3,000. The predicted targets were much higher than the actual targets^92^. Also, CK2 phosphorylates a few substrates that do not have the consensus sequence. Thus, target validation is essential in determining the bona-fide substrates of CK2. It also helps target interactions where alternate kinases cannot nullify the drug's effect. Approaches like phospho-proteomics have helped determine the number of targets this kinase affects.

Chonjowski et al. developed a method for validating genuine targets of CK2, specifically by taking advantage of the co-substrate specificity of CK2. The co-substrate specificity of CK2 means that the kinase can use either adenosine triphosphate (ATP) or guanosine triphosphate (GTP) to phosphorylate its substrates. Briefly, an analog of GTP - guanosine 5'-[γ-thio]triphosphate (GTPγS) is incorporated into the cell suspension. Other endogenous kinases do not use this substrate. Thus, substrates phosphorylated with CK2 are identified by the presence of ‘PγS’ using Mass Spectrometry. Further, validation of CK2 targets with co-immunoprecipitation is possible with substrate-specific antibodies and pull-down assays. This method allows the use of versatile biological samples and helps capture phosphorylation reactions while the cells are still alive ^31;93^.

Inhibition of CK2 kinase activity by ATP competitive inhibitors does not alter the phosphorylation of all its targets. Changes in phosphorylation levels of CK2 targets by two CK2 inhibitors, Silmitasertib and GO289, were different. Both inhibitors have very low off-target activity and have an almost overlapping set of affected CK2 phosphorylation targets. However, the extent of reduction in phosphorylation of its targets is very different between these two. Both inhibitors bind the same site on CK2α and cause very different effects. This difference could be attributed to the ability of CK2 to identify its targets under various conditions ^18; 94^.

To identify substrates exclusively phosphorylated by CK2, inhibitor-resistant mutants of CK2 were created. The kinase is known to phosphorylate only 20% of the substrates containing the minimal recognition motif. With the mutants and subsequent use of inhibitors, the expanse of substrates was estimated in C2C12 cell lines. Studies using lysates do not consider the temporal and spatial factors for CK2-substrate interactions. Instead, these studies used the stable isotope labeling using amino acids in cell culture (SILAC) approach. This allowed the study of interaction when the cells performed their normal function. The disparity was observed within popular CK2 inhibitors, with only a proportion of inhibitors showing dose-dependent inhibition of CK2 activity ^95^.

#### 3.1.2. Function of subunits

CK2 has catalytic subunits, CK2α and CK2α’, as well as regulatory CK2β subunits. The enzyme functions as a holoenzyme, and each subunit can also work in isolation^37^. Individual subunits of CK2 seem to play diverse roles exclusive to the holoenzyme form. There are many instances where individual subunits of CK2 perform distinct functions^96^. Inhibition of a particular subunit or its depletion in cellular compartments affects the expression and activity of other subunits. Also, by performing subunit knockout, the extent of the contribution of individual subunits was determined. This approach is helpful in the creation of subunit-specific drugs. In most cases, the abrogation of catalytic subunits impacts the expression and function of other subunits. These are explained using the following examples.

A coordinated regulation mechanism between the three holoenzyme subunits was observed during skeletal muscle differentiation. The mechanism involved regulating early Myogenic Regulator Genes such as myoblast determination protein 1 (MyoD) and myogenin, followed by the expression of muscle-specific genes such as caveolin-3, troponin, and myomixer. The holoenzyme's individual subunits and catalytic activity also coordinated plasma membrane fusion and the shutting of embedded proteins. In individual knockouts of subunits in C2C12 cells with CRISPR/Cas9, the deletion of one subunit affected the expression of other subunits, demonstrating their interdependence. Deletion of the CK2α subunit downregulated CK2β expression, whereas deletion of CK2β subunit upregulated CK2α expression. However, deletion of CK2α’ did not alter the levels of the other two units. In knockouts of CK2α and CK2β, MyoD expression was reduced and abrogated, respectively, whereas the deletion of CK2α’ did not have an effect. The reduction of MyoD expression in CK2α deletion was due to the downregulation of CK2β in these mutants. In CK2β null mutants, the expression of this early myogenic regulatory factor (MRF) was not activated until the re-induction of the subunit in CK2β knockdown C2C12 cells already expressing low levels of MyoD. With increasing expression of CK2β in these cells, the level of MyoD increased, which was also reflected as an increase in its mRNA at the transcription level. This indicates that the CK2β with other epigenetic modifiers activate the expression of MyoD. The level of MyoD expression was not affected by varying the catalytic activity of CK2 by pharmacological inhibition or changes in the expression of CK2α and CK2α’ subunits. During the later stages of myogenic differentiation, the levels of MyoD reduce, whereas the expression of muscle-specific genes increases in WT C2C12 cells. Here, pharmacological inhibition of kinase activity in CK2α’ knockout mutants reduced the expression of these markers. This evidence indicates the role of the other catalytic subunit CK2α in the expression of muscle-specific markers in later stages. The role of CK2α’ during myotube formation was indispensable. With the deletion of this subunit, there was a reduction in the number and size of myotubes compared to WT. Exogenous CK2α’ is sufficient to rescue reduced fusogenic activity of muscle fibers because of CK2 deletion^78^.

One of the two catalytic subunits, either CK2α or CK2α’, is more critical in some biological processes. This is the case during skeletal muscle development and homeostasis. Here, the activity of the CK2α subunit is crucial^97^. Skeletal muscle specific CK2α knockout mice were produced by breeding floxed mice with human skeletal actin Cre reporter mice. The grip strength of the CK2α knockout mice was decreased compared to the controls, and the difference was more significant in older mice. CK2β expression levels were significantly reduced in the CK2α knockout mice, but expression of CK2α’decreased slightly. Phospho-Akt1 serine 129 and 473 proteins were upregulated in the knockout mice. There was no difference in gastrocnemius and soleus muscles between the CK2α knockout mice and the controls with Hematoxylin and Eosin staining. The muscle sections from the knockout mice had higher levels of centralized nuclei than the controls, indicating their higher propensity for muscular regeneration. The soleus fibers of the knockout mice had lower levels of slow type I muscle fibers but increased levels of fast type II fibers compared to the control. This evidence suggests that CK2α is essential in skeletal muscle regeneration. CK2α knockout mice had elevated expression of several mitophagy markers, including sequestosome-1(SQSTM1), optineurin (OPTN), and microtubule associated protein 1 light chain 3 beta (MAP1LC3B-II). The upregulation of these markers suggests that autophagic processes were increased in the absence of CK2 catalytic activity. These mice also had decreased levels of mitochondrial genomic DNA. Knockout of CK2α also impaired neuromuscular function. The expression of various proteins essential to skeletal muscle function was aberrant ^98^. Thus, deleting one catalytic alone subunit was sufficient to hamper the process.

Knockout of CK2 catalytic and regulatory subunits in myogenic C2C12 cells had very different effects. With the knockout of CK2α and CK2α’ subunits, there was a substantial reduction in phosphorylation of predicted CK2 targets (18 out of 24). Expression of the CK2β subunit was reduced with this deletion. One possible explanation could be an increased rate of degradation of CK2β. Phosphorylation was upregulated in some of the non-CK2 phosphorylation targets, which could be explained by changes in the expression and activity of other kinases in the absence of CK2 catalytic subunits. In the knockout of CK2β subunits, there was a proportion of reduction of phosphorylation that was slightly lower than that of the catalytic units (9 out of 15). Here, the catalytic subunit expression was increased compared to the wild type. For some of the CK2 substrates, such as Cdc37 (pS13), the knockout of CK2 did not affect phosphorylation. However, for the substrate, Akt S129, the phosphorylation was drastically reduced to the extent it was almost absent with the knockout of either the catalytic or regulatory subunit. These differential effects on substrates shed light on the function of CK2 subunits as compensatory to each other in some cases or the strict requirements of holoenzymes in others. Changes in the phosphorylation status of predicted CK2 targets and non-targets based on sequence prediction indicated that the consensus sequence alone is not sufficient or essential for target identification and phosphorylation to occur ^31^.

### 3.2. Small molecular inhibitors of CK2 and their mechanism of action.

**Figure 1:**
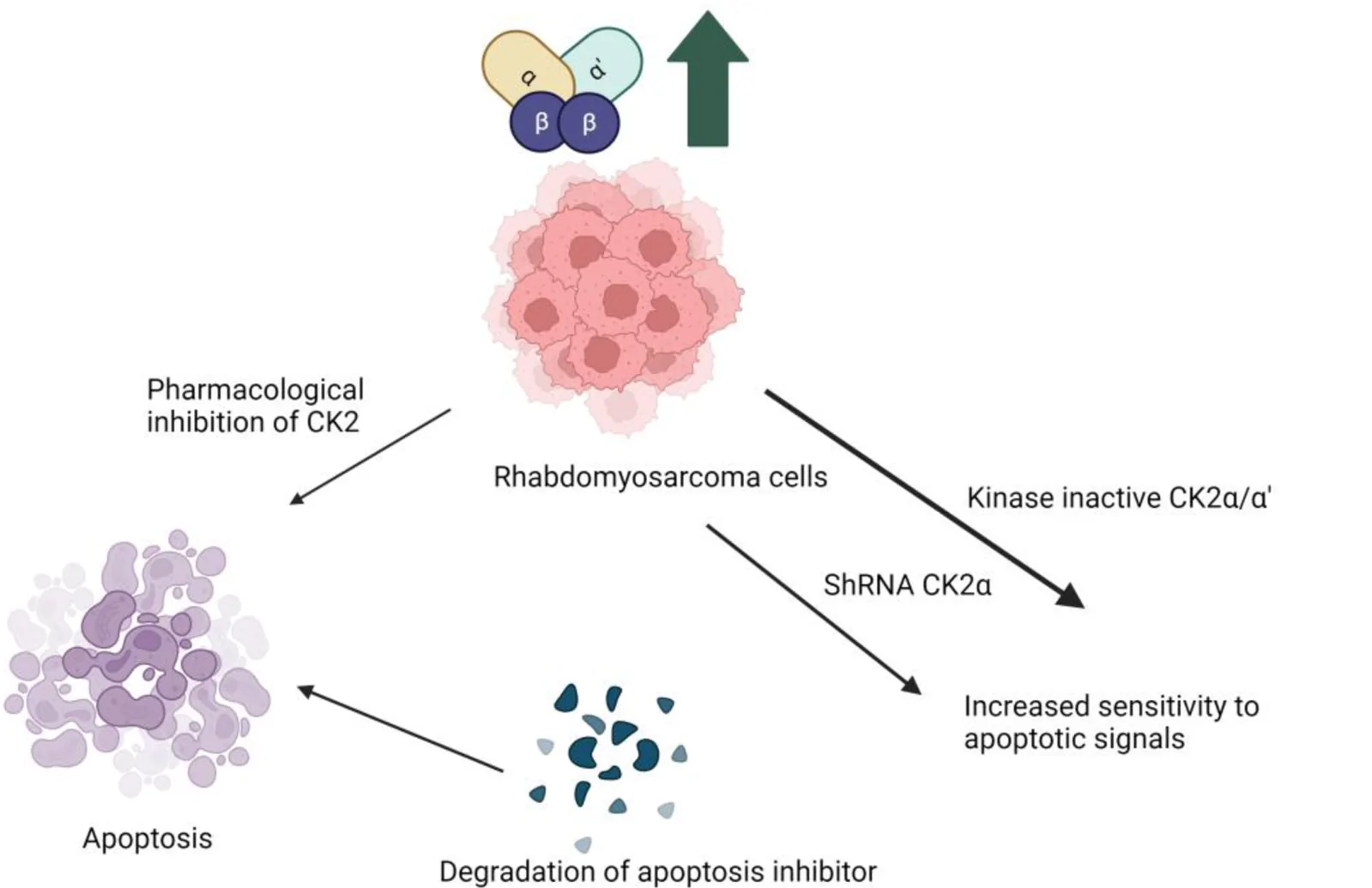
Implication of CK2 in Osteosarcoma and effect of inhibition of CK2 kinase activity. Inhibition of CK2 makes the cells more sensitive to Apoptotic signals. (Image is created with Biorender.com)

A critical application of small molecule inhibitors of CK2 is within the treatment of cancer^99; 100^. For example, osteosarcoma, a bone malignancy that originates in the osteoblasts, is one such cancer in which these inhibitors are an effective treatment. In osteosarcoma, CK2 is overexpressed. In human cloned osteosarcoma 143B cell line CK2α and CK2β are upregulated, compared to healthy osteoblasts and MSCs. The osteosarcoma cells treated with the CK2 inhibitor Silmitasertib suppressed growth and proliferation and increased apoptosis. Interestingly, the proliferation and differentiation of MSCs were unaffected. The siRNA-induced knockdown of the CK2α and CK2β subunits *in vitro* suppressed the growth and proliferation of osteosarcoma cells. The 143B osteosarcoma cells were xenografted into mice and treated with Silmitasertib. Similar to *in vitro* findings, Silmitasertib inhibited the proliferation of the tumor cells and, therefore, constrained the growth of the tumor. These findings support that Silmitasertib is a potential therapeutic for osteosarcoma. It inhibits CK2, which induces the apoptosis of sarcoma cells while having little to no effect on MSCs ^74^. The implication of CK2 inhibition in osteosarcoma is explained in Figure 1.

Rhabdomyosarcoma, a cancer initiated in the skeletal muscle. Treatment of rhabdomyosarcoma is another example of the therapeutic implications of CK2 inhibitors. CK2 is upregulated in rhabdomyosarcoma cells. Current research on the pathophysiology of rhabdomyosarcoma uses cloned human tumor cell lines JR1 and Rh30. When these cell lines were treated with the CK2 inhibitor 5,6-dichloro benzimidazole (DRB), the proliferation of pro-apoptotic signals commenced, such as cytochrome c and Smad/DIABLO. This caused widespread cell death. JR1 and Rh30 cells transfected with either short hairpin RNA targeted to CK2α or kinase inactive CK2α/CK2α’ led to heightened sensitivity to pro-apoptotic signals, due to the suppressed CK2 activity. Inhibitors of CK2 have the potential as therapeutics to prevent the growth and metastasis of rhabdomyosarcoma cells^73^. Here, inhibition of kinase activity and knockdown of α/α’ subunit increased apoptosis.

Additionally, research supports the utility of CK2 inhibition in treating rheumatoid arthritis (RA). In one study, human primary CD4+T lymphocytes isolated from patients diagnosed with RA were treated with the CK2 inhibitor Silmitasertib. Cellular responses in Th1 and Th17 cells were suppressed compared to controls. However, the Th2 cellular activity was enhanced. Th1 cells secrete proinflammatory factors, such as TNF-α and IL-2, while Th2 cells secrete anti-inflammatory factors, such as IL-3, IL-4, and IL-5^101^. In the same study, the collagen-induced RA mouse model, the effects of CK2 inhibition were similar ^102^. Mice were treated with Silmitasertib via ingestion. The Th1 and Th17 cell responses were inhibited, and their RA symptoms were reduced ^57; 102^.

**Figure 2:**
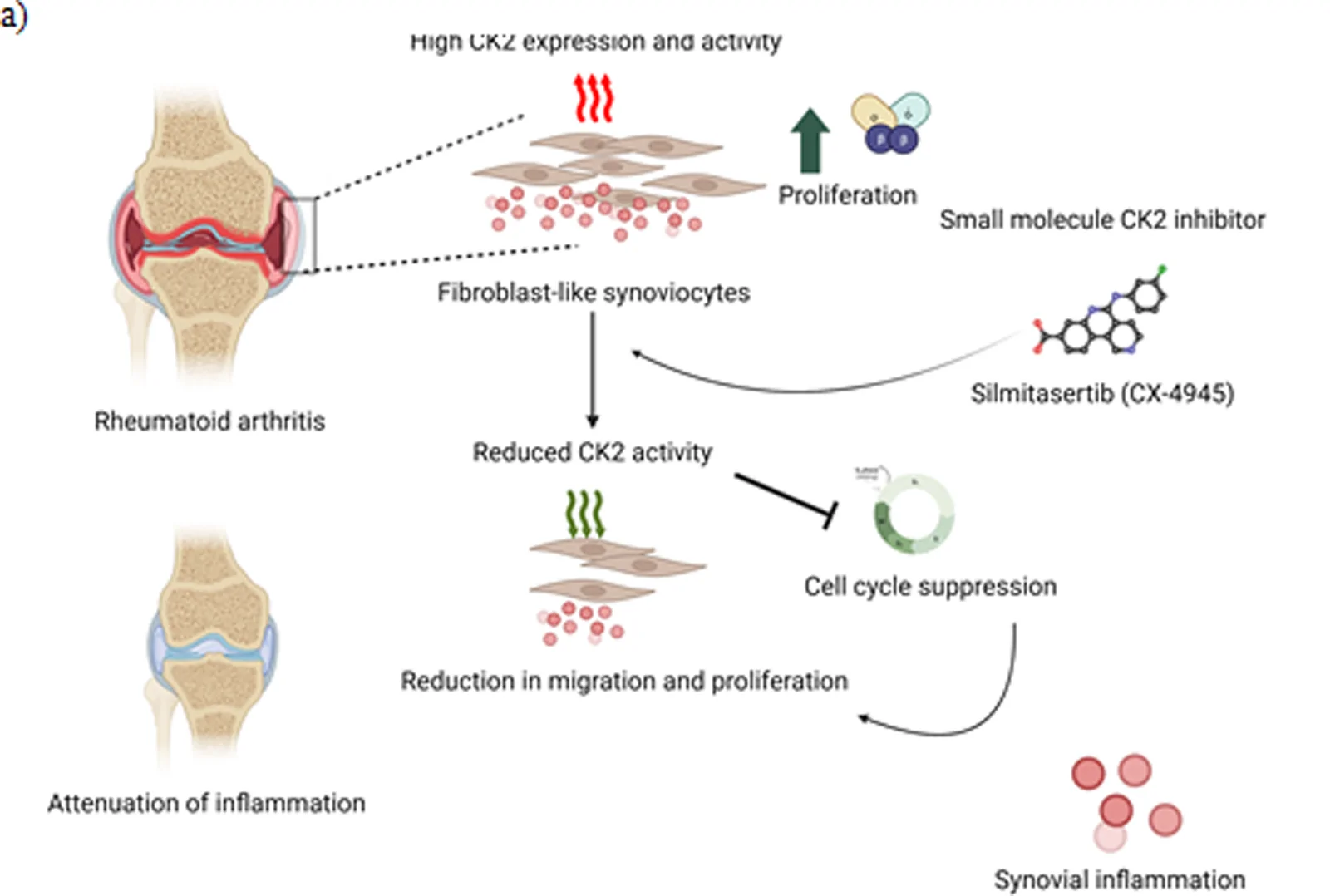

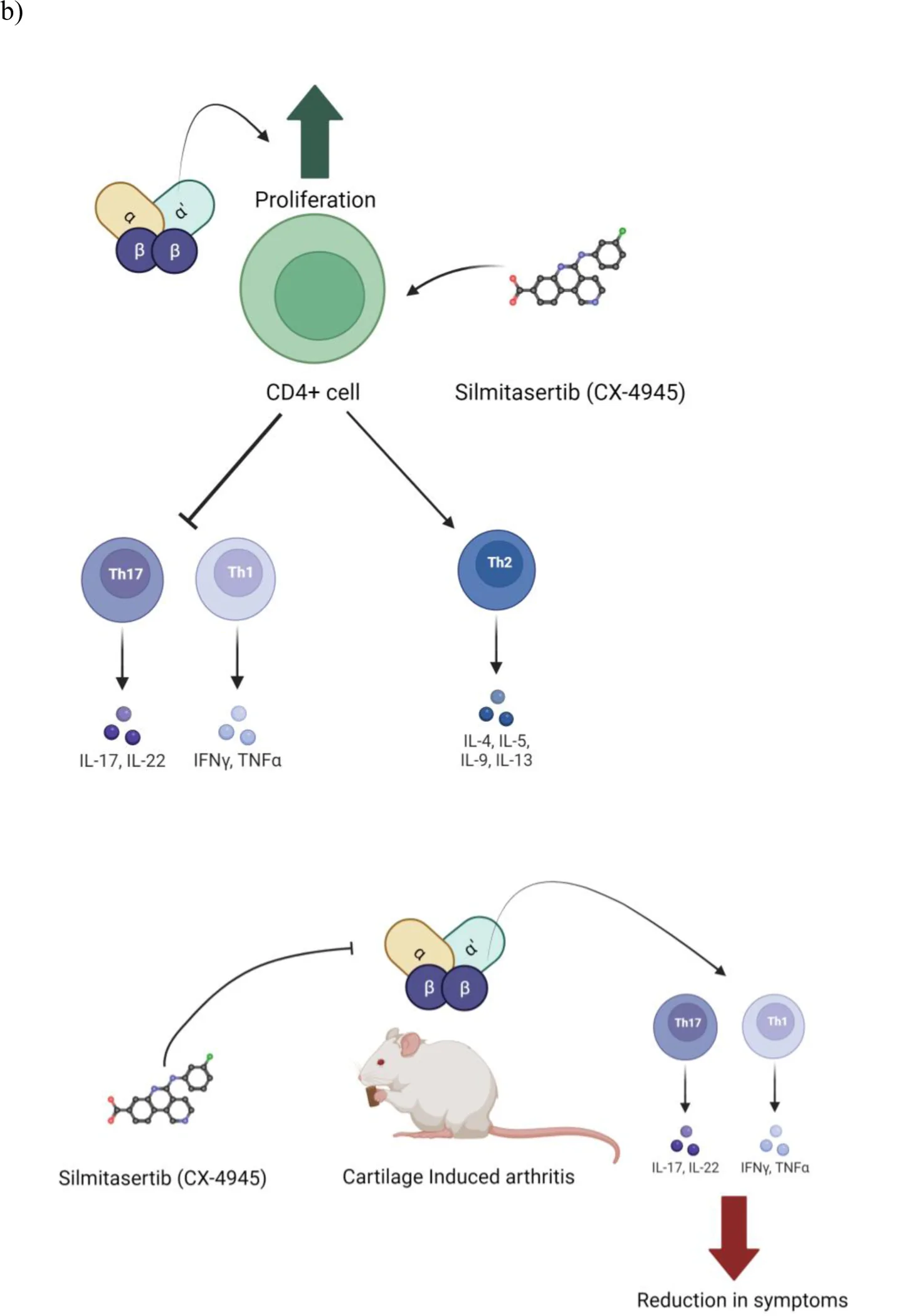
Implication of CK2 in RA and effect of inhibition of CK2 kinase activity. In RA, the expression of CK2 is upregulated, contributing to increased inflammation by various mechanisms. a) Inhibition of CK2 in fibroblast-like synoviocytes reduced their proliferation and hence reduced the synovial inflammation. b) In RA, CD4+ cells treated with CK2 inhibitors had a reduction in activation of Th1 and Th17 T cell response, and it activated Th2 type cell response. Inhibition of CK2 in CD4+ cells reduced their proliferation, and hence, Th1 and Th17 T cell response was activated. These effects were seen in *in-vivo* conditions as well. (Image created with Biorender.com)

Another important cellular component in inflammatory pathologies is the primary fibroblast-like synoviocyte (FLS). These are abundant in synovial tissue and function to regulate synovial fluid as well as extracellular matrix ^57^. In one study, primary FLSs from patients diagnosed with RA showed higher levels of CK2 expression and activity than primary FLSs from patients diagnosed with OA, the control. Stimulation of the RA primary FLSs with Silmitasertib suppressed the cell cycle by inhibiting CK2, a key component in regulating the cell cycle. Further, the drug’s inhibition of CK2 decreased migration and proliferation of RA-FLSs, ultimately attenuating symptoms of RA, such as inflammation and joint pain ^103^. Figure 2 summarizes *in-vitro* and *in-vivo* findings from these two studies.

The two most promising CK2 inhibitors include small molecule Silmitasertib and peptide inhibitor CIBG-300^29^. Both are under clinical investigation for their use in cancer treatment. The ATP-competitive CK2-specific small molecule inhibitor exploits the differences in this kinase's ATP-binding domain with others. The ATP-binding domain in the CK2α subunit is small compared to other kinases of this family.

Silmitasertib is highly specific for CK2 amongst small molecule inhibitors of CK2, but the drug is not without adverse effects^98^. The inhibitor caused abnormal alternative splicing in CK2 independent manner^104^, the CK2α transcript was affected as well ^97^. In Cholangiocarcinoma, the drug induced methusis and caused cell death in a CK2-independent manner. In pancreatic ductal adenocarcinoma (PDAC), the responsiveness to Silmitasertib was tested in different cell lines. Expression of CK2 varied in the cell lines tested, although it was elevated in all of them compared to non-neoplastic cells. Elevated expression of CK2 in tumor cells Responsiveness to Silmitasertib was not correlated to the extent of CK2 upregulation within the cell lines tested^105^. Additionally, inhibition of total CK2 activity within the cell can cause uncontrollable events in non-tumor cells^105; 106; 107^.

Silmitasertib also has off target effects. Silmitasertib inhibited dual-specificity tyrosine phosphorylation-regulated kinase 1A (DYRK1A) and glycogen synthase kinase-3β (GSK3β) kinases^108^. Off-target effects are also seen in the case of inhibition of Cdc2-like kinases (Clks). Furthermore, the efficacy of Silmitasertib in cancer is also lower than that of several other inhibitors of CK2, which can be ascribed to its moderate selectivity^99^. The off-target activity of Silmitasertib is reported in the literature^86^. Because Silmitasertib has many off-target effects, a more specific molecule based on the drug’s structure is now under development^108^.

The utility of CK2 inhibition by Silmitasertib for treating a non-neoplastic disease- osteoporosis was tested. Here, the effect of sub-cytotoxic concentrations of Silmitasertib was tested on osteoblast and osteoclast differentiation. It inhibited differentiation of Bone Marrow-derived Macrophages (BMM) into osteoclasts through RANKL activation. Inhibition of CK2 reduced phosphorylation of Akt, which caused downregulation in the expression of the transcription factor Nuclear Factor of Activated T Cells 1 (NFATc1), an effector of the Akt signaling cascade. Inhibition of CK2 also reduced RANKL-induced formation of tartrate-resistant acid phosphatase (TRAP), positive multinucleated cell formation, and TRAP activity, which is essential for osteoclast formation. On the other hand, BMP-2-mediated differentiation of myoblastic C2C12 cells into osteoblasts was enhanced with CK2 inhibition. Alkaline Phosphatase (ALP) activity and nuclear smad1/5/8 accumulation increased with increasing Silmitasertib concentration in differentiating cells. There was also an increase in Extracellular receptor Kinase (ERK) 1/2 phosphorylation. Thus, inhibition of CK2 accelerated osteoblast differentiation via ERK1/2-Smad signaling axis^109^.

Allosteric inhibitors bind at a site other than the enzyme's active site. They cause a change in the active conformation of the enzyme, which hampers substrate binding. Thus, they are not competitive for the substrate of the enzyme^110^. Allosteric inhibitors of CK2 are non-ATP competitive^111^. Allosteric inhibitors of CK2 can bind to three different sites^112^. Most effective inhibitors bind at the α and β unit interface^113^. This approach retains the activity of the catalytic subunits and hence only affects the phosphorylation of CK2β dependent substrates ^29^. The regulatory β subunits act as docking sites for a few CK2 substrates and protect the catalytic subunits from degradation. The other two sites where allosteric inhibitors can bind are the αD pocket, the linker region in the ATP binding site ^114^, and the αC helix region, which is rich in glycine. Detailed descriptions of various CK2 inhibitors and their strategies can be found in the current literature^29^. The structure-activity relation of CK2 inhibitors is reviewed in literature^29; 115; 116; 117^. Many of these are either not highly specific or have low cell permeability. Some compounds known to inhibit CK2 do not show cytotoxicity in cancer cell lines. However, their utility in treating musculoskeletal disorders is worth investigating. In these diseases, re-directing pathways involving deregulated CK2 is more critical than causing general cytotoxicity.

Further studies are needed for clinical translation of these inhibitors. Examples of small molecular CK2 inhibitors are included in Table 1. Based on kinase panel assay, their selectivity in inhibiting CK2 is mentioned as CK2 selectivity. The pre-clinical efficacy is described for each one of these.

It is an effective strategy to target CK2 with other kinases that are either phosphorylated by it or act on the same substrate. Here, Bromodomain-Containing Protein 4 (BRD4) is a kinase that, along with CK2, is highly expressed in several different types of cancer. BRD4 is a transcriptional and epigenetic regulatory factor that belongs to the bromodomain and extraterminal (BET) family of chromatin adapter proteins. Its localization and stability are regulated by its phosphorylation by CK2. The use of inhibitors of BRD4 in cancer treatment is limited due to drug resistance^141^. In lung adenocarcinoma, the mechanism of drug resistance against BRD4 inhibitors was CK2 dependent^142^. A dual inhibitor for CK2 and BRD4 was more effective than targeting CK2 alone. Similarly, proviral integration of Moloney virus-1 (PIM) kinases and CK2 interacts with Myc^143; 144^. These two kinases also have functional and structural similarities. In diseases with high expression of PIM and CK2, it was more effective to target both kinases. Both kinases are upregulated in hypoxic conditions in tumors^145^. Kinase SR-protein-specific kinase 1 (SRPK1) is involved in angiogenesis and overexpressed in several cancers. SRPIN803 inhibited SRPK1 and CK2 together. Its ophthalmic application was also studied. TNIK and DYRK1 help maintain the phosphorylation of key regulators in cancer stem cells. Compound 108600 inhibited the TNIK, DYRK1, and CK2. However, there was also an allosteric change in the CK2 α subunit to inhibit holoenzyme formation^110^.

The synthetic peptide CIBG-300 is effective in the treatment of cervical cancer. It is currently in phase 1 and 2 clinical trials^151^. It was also found to be anti-angiogenic^152^. The drug is derived from a cyclic nine-mer peptide. This peptide inhibited phosphorylation of human papillomavirus type-16 E7 (HPV-16 E7) oncoprotein phosphorylation in a phage display assay. Upon conjugating with cell-penetrating peptide Tat (amino acids 48–68) to increase the cell permeability of the peptide, it inhibited phosphorylation of the HPV-16 E7 oncoprotein by CK2 and induced apoptosis in cell cultures^42^. This peptide was then studied in different types of cancer^153^. CIGB-300 interacted more with catalytic subunits and interacted less with the regulatory subunit. Also, it responded with the CK2α’ more than the CK2α subunit^43^. The target of this peptide was B23/nucleophosmin, a nucleolar protein that selectively modulates specific genes. It affected protein synthesis, energy metabolism, and biogenesis of ribosomes^141^. These findings have led to a challenge in the defining mechanism of action of CIBG-300. Previously termed as CK2 inhibitor, its specificity in targeting selected phosphosites rather than generalized inhibition is getting attention.

Inhibitors with a higher specificity can target specific subunits or forms of the CK2 holoenzyme. Some of them, like CIBG-300, are capable of inhibiting selective phosphosites. They are tested for their use in cancer treatment as well as treatment of other diseases. These are listed in Table 3.

## 4. Targeting CK2 interactions with its substrates - Novel approach

Targeting CK2-substrates interaction critical for pathogenesis for musculoskeletal disorders can be done to engineer metabolic pathways to a healthy state. Interfering peptides inspired by the structure of the CK2 substrate can be used for this purpose.

Such biomimetic peptides are designed based on Protein-protein interactions (PPIs). PPIs are essential to cellular functioning, and any dysfunction could represent an opportunity for specific targeting^157^. The nature of these interactions is being established using cutting-edge computational methods paired with existing knowledge. These studies aid in the design of drugs targeting very specific PPIs implicated in disease pathology^158^. Peptides and monoclonal antibodies have been widely used to produce new therapeutics to target extracellular PPIs. Still, the development of peptides targeting intracellular PPIs has been far more limited due to the challenges of maintaining the peptide’s stability, administration, and bioavailability. Interfering peptides can interfere with PPIs and have been the focus of increasing attention by researchers. The large contact surface associated with PPIs has previously been an obstacle in the development of therapeutics, but interfering peptides have been found to interact more efficiently with large protein surfaces^159^. Peptides offer several advantages when used as therapeutics compared to small molecular drugs. First, they can cover more surface area in the PPI they are inhibiting. Then, their physical properties are easily modifiable based on their application. Moreover, they can be designed to be highly selective and can cover more surface area as inhibitors. Also, they have fewer toxicological effects^160^. Importantly, peptides can be developed to penetrate specific plasma membranes, which aids in targeting them to specific intracellular compartments, a long-standing challenge in therapeutic development^161; 162; 163; 164^. Several studies prove that interfering peptides alter cellular processes ^156; 165; 166; 167^. Peptides can be produced in several ways, the simplest being procuring naturally occurring peptides^168^. Another method is to develop large numbers of peptides synthetically and then screen them to evaluate their therapeutic potential. A third method is to use peptides corresponding to fragments at the ends of the PPIs^169^.

### 4.1. Disease Modifying Peptide Drugs- CK2.1 and CK2.3

**Figure 4:**
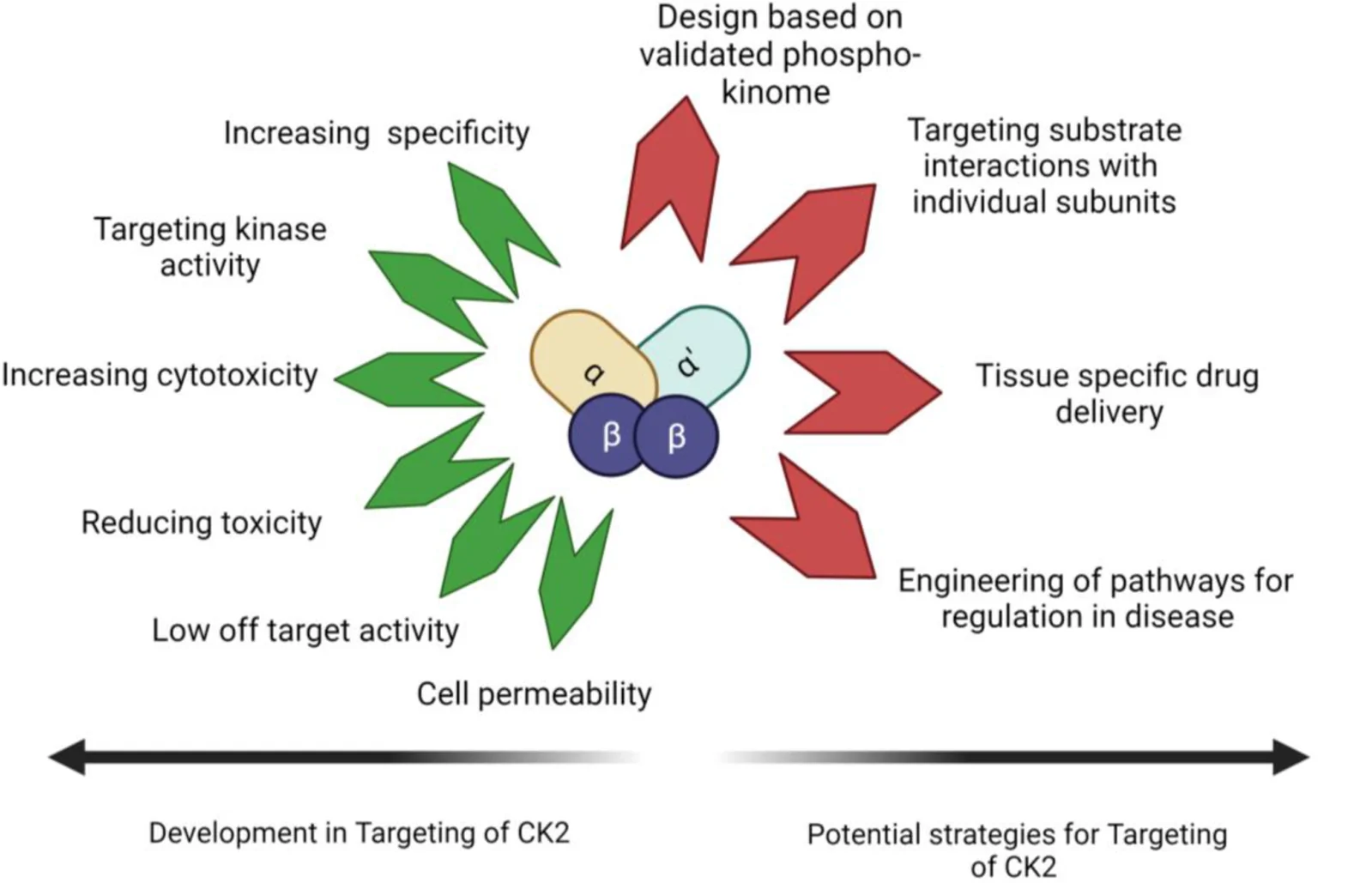
CK2 inhibitors have been designed with increased specificity for CK2, reducing off-target effects and increasing cell permeability. CK2 inhibitors have been found to increase cytotoxicity in anti-cancer drugs. With knowledge of the phosphosites of CK2 and the role of CK2 subunits and molecular forms, future CK2 inhibitors can be designed to target pathology-specific substrate interactions (Image Created with BioRender.com)

One of the most integral ways in which CK2 regulates the function of the musculoskeletal system is through the Bone Morphogenetic Protein (BMP) Signaling Pathway. The BMP signaling pathway regulates chondrogenesis and osteogenesis and maintains connective tissue homeostasis. The BMP-2 ligand interacts with BMPRIa and BMPRII receptors to activate the BMP pathway. CK2 interacts with the BMPRIa receptor in the absence of the BMP-2 ligand. Upon binding the BMP-2 ligand, the BMPRIa receptor undergoes heterodimerization with the BMPRII receptor. Formation of this complex releases of CK2. The release of CK2 initiates the subsequent phosphorylation of downstream signaling, which can cause the differentiation of MSCs into adipocytes, osteoblasts, and chondrocytes, depending on the pathway initiated. However, controlling the cell's fate via the signaling cascade activated by BMP2 is impossible. This led to the design of interfering peptides activating specific branches of the BMP pathway ^170^. Three BMPRIa mimetic peptides were synthesized to initiate the BMP signaling pathway without BMP2. These peptides contained one of three CK2 phosphorylation sites on BMPRIa (SLKD, SYED, or SLYD). At the N-terminus, there is the Antennapedia Homeodomain sequence for cellular uptake. Flanking the CK2 phosphorylation motif, there is a BMPRIa homologous sequence. These peptides are named CK2.1, CK2.2, and CK2.3 based on the phosphorylation site they contain^171^. CK2.1 induced chondrogenesis in *vitro* in mouse MSC line C3H10T1/2 and in *vivo* in the Destabilized Medial Meniscus (DMM) mouse model. CK2.1 increased proteoglycan synthesis and elevated levels of collagen type II without causing chondrocyte hypertrophy, a frequent occurrence with BMP2-induced signaling^172; 173^. Rabbit MSCs were treated with a bilayer peptide-loaded scaffold consisting of CK2.1 coated β-glycerophosphate/chitosan, which resulted in chondrogenesis and osteoblastogenesis. This technique could be beneficial for treating articular osteochondral defects, common in OA patients, involving damage to the cartilage and underlying bone^23^. CK2.2 induced both adipogenesis and Osteogenesis, while CK2.3 induced Osteogenesis. CK2.3 would be preferred in treating OP and other disorders that reduce bone mass and mineral density because it does not simultaneously induce adipogenesis, as does CK2.2^172^. CK2.3 activates ERK phosphorylation by preventing CK2 binding to the BMRPIa-BMPRII receptor complex. CK2.3 increases bone area, bone mass, and mineral density ^174^. In C2C12 cloned mouse myoblast cells, CK2.3 decreased osteoclastogenesis, increased osteoblastogenesis, and increased mineralization compared to controls. CK2.3 has been tested in an aging mouse model. *In vivo*, injections of CK2.3 via calvarial injection in mice increased bone area, density, and growth^171^. In 6 to 9-day-old C57BL/6J mice injected with CK2.3 via the calvaria and 8-week-old mice injected via the tail vein, mice showed increased bone formation and increased bone mineral density. At the same time, osteoclast activity appeared to be suppressed, indicating CK2.3 has the potential to alleviate the symptoms of OP by simultaneously enhancing osteoblast activity and inhibiting osteoclast activity, inducing bone growth and repair^175; 176; 177^. The principle of design of BMPRIa biomimetic peptides CK2.1 and CK2.3 and their effects are summarized in Figure 4.

## 8. Discussion

Inhibition of CK2 is proving to be highly successful in treating cancer. In musculoskeletal disorders, the influence of metabolic disorders is a research focus. The kinase CK2 is highly involved in the regulation of cellular metabolism. In rheumatoid arthritis, elevated CK2 activity promotes the inflammatory pathways. In osteoarthritis, a reduction in the activity of CK2 reduces the viability of chondrocytes. In osteoporosis, the imbalance in BMP signaling is due to the dysregulated function of CK2. During fracture healing, aging and senescence cause defective bone formation. CK2 is essential in cell survival and helps the cell counter senescence. Studying the role of CK2 in these diseases will demystify several mechanisms involved.

The pathogenesis of most musculoskeletal disorders has a component of dysregulated cell differentiation, senescence, and inflammation. Causing cytotoxicity is not always the best course of action for these disorders. Instead, correction of underlying biochemical imbalances is an effective treatment. The role of CK2 as the lateral player makes it an ideal protein target for these disorders.

The evolution of small molecular inhibitors of CK2 is mainly focused on their use as cancer therapeutics. Improved specificity and cell permeability are the two main achievements. These inhibitors are commonly used in combination with targeted therapy. However, their use in treating non-neoplastic disorders like musculoskeletal disorders is not common. Also, targeting the kinase activity is only sometimes the most effective method for this group of disorders. The entire phosphoproteome of CK2 is still being experimentally validated. Targeting of redundant interactions and false positive substrates hence stands as a possibility. The peptide drugs target the identified interaction between CK2 and BMPRIa. These are also seen to be highly cell-permeable. More investigation into their tissue-specific drug delivery and toxicity needs to be done. Evidence of their ability to drive the differentiation of MSCs into different lineages opens many possibilities. Drugs highly specific to kinase substrate interaction can be a molecular engineering tool. It can direct the molecular pathways towards the desired lineage. Targeting the pleiotropic kinase CK2 in this manner is an emerging prospect for designing a new generation of drugs. Figure 4 illustrates the current advances in CK2 inhibitors and the aspects to be addressed by upcoming drugs targeting CK2-substrate interactions.

## Figures and Tables

**Figure 3:**
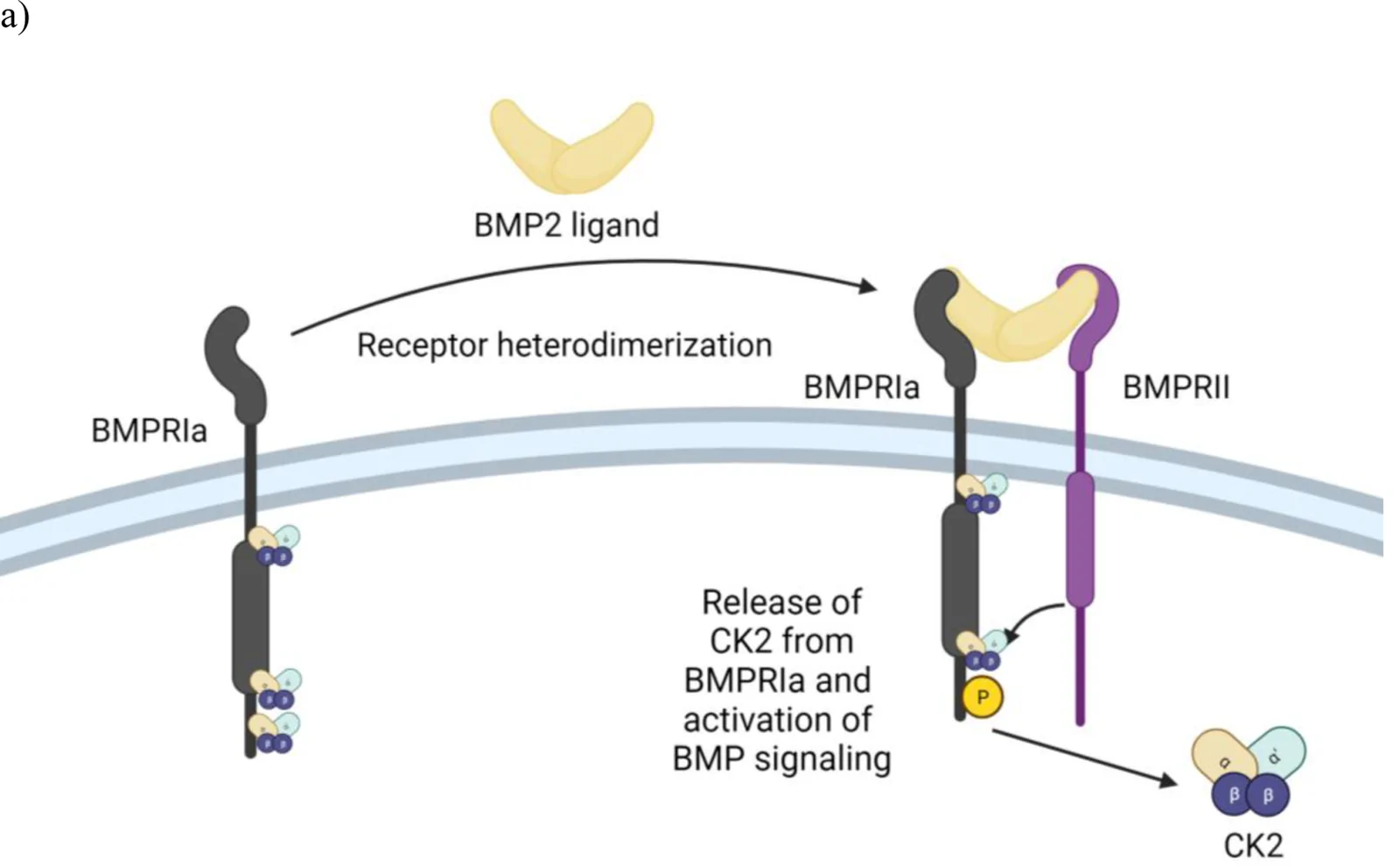

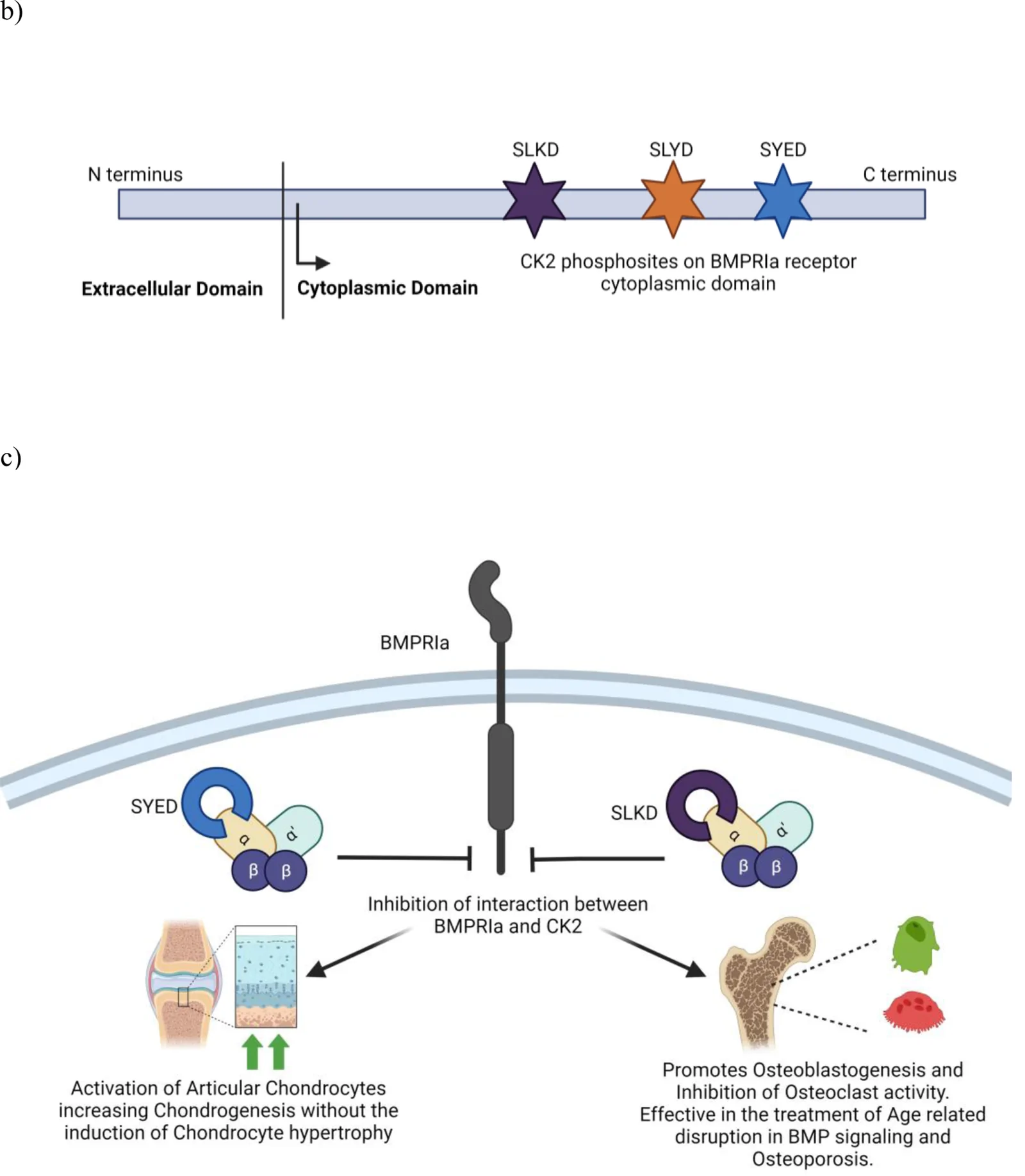
Inhibition of the interaction between CK2 and BMPRIa is an effective strategy for treating Osteoarthritis and Osteoporosis. a) Stimulation with BMP2 ligand causes heterodimerization of BMPRIa receptor with BMPRII. Release of CK2 from BMPRIa and its phosphorylation by BMPRII activates the BMP signaling pathway. b) BMPRIa receptor has three phosphosites on the cytoplasmic/intracellular domain. These phosphosites were validated using CK2 mutants. c) BMPRIa mimetic peptides containing phosphosite sequences corresponding to ‘SYED’ activate articular chondrocytes to repair osteoarthritic lesions in knee articular cartilage. The BMPRIa mimetic peptide containing phosphosite sequence corresponding to ‘SLKD’ corrects the BMP pathway in senile mice. It helps regain balance between Osteoblastogenesis and Osteoclastic activity, which is affected in Osteoporosis. (Image created with Biorender.com)

**Table 1.** Small molecular inhibitors of CK2

Name	CK2 selectivity	Efficacy	Reference
Silmitasertib (CX-4945)	Selective at low concentrations. However, at nanomolar concentrations, it inhibits other kinases, such as DYRK1A.	Currently, it is the most selective CK2 inhibitor in clinical use and has minimal toxicity. Promotes apoptosis while inhibiting the PI3K/Akt signaling pathway and the cell cycle progression. Inhibits angiogenesis.	^109; 118; 119; 120^
dimethylamino-4,5,6,7–1H-tetrabromobenzimidazole (DMAT)	Selective for CK2 but inhibits DYRK1A	Induces DNA double-strand breaks. Promotes apoptosis. Inhibition of aldosterone, DHEAS, and androstenedione secretion in adenocarcinoma cell line H295R.	^27; 121; 122^
Emodin	Moderately selective	Natural anthraquinone derivative extracted from rhubarb. Inhibits CK2.	^123; 124; 125^
Quinalizarin	Fairly selective	Promotes apoptosis in HEK-293 and Jurkat cells.	^126; 127^
IC20	Extremely selective for CK2α	Not cytotoxic in cancer cells	^128; 129; 130; 131^
SGC-CK2–1	Selective	Used as a cellular probe to investigate the intracellular function of CK2. Low inhibitory effect on the proliferation of most cancer cells, effective against a small subset of cancer cells.	^132; 133^
Tetrabromocinnamic Acid (TBCA)	Selective	Derived from 4,5,6,7-tetrabromobenzotriazole (TBB). It is more potent than TBB. Does not inhibit DYRK1 like TBB. Promotes apoptosis in Jurkat cancer cells. Suppresses platelet aggregation/secretion and the cell cycle progression in prostate cancer cells.	^134; 135^
4-(6,8-Dibromo-3-hydroxy-4-oxo-4H-chromo-2-yl)-benzoic acid (FLC26)	Mildly selective	Causes a significant increase in apoptosis in PANC-1 cells	^136; 137^
3,8-dibromo-7-hydroxy-4-methylchromen-2-one (DBC)	Poorly selective	Induces apoptosis in Jurkat cells.	^138^
CAM4066	Poorly selective	Acts on the αD region and ATP-binding sites of CK2. The moiety bound to the ATP binding site forms a hydrogen bond with Lys68 and two water molecules. The moiety in the αD site interacts with Pro159 and a conserved water molecule. The linker creates a network of hydrogen bonds. Poor permeability. Methyl ester derivative pro-CAM4066 has better permeability.	^73; 114; 139^
CAM4712	Highly selective	Anti-proliferative	^110; 114^
GO289	High selectivity	Extremely selective, ideal for clinical use. Inhibition of the phosphorylation sites of multiple clock proteins and suppressed the growth/proliferation of cells of a diverse array of cancers. CK2α and CK2α′ are the primary targets of the drug.	^116; 120^
HY1-Pt	Highly selective. Derived from Silmitasertib. Overcomes cisplatin-induced resistance.	Reversed cisplatin-induced drug resistance. Suppresses DNA damage repair in cancer cells. It also inhibited the Wnt/beta-catenin signaling pathway while activating the mitochondrial apoptosis pathway. It displayed no toxicity to healthy hepatocytes and could be used as a therapeutic for NSCLC.	^108; 140^

**Table 2:** Multitarget inhibitors of CK2

Name	Co-Target(s)	Clinical Efficacy	Reference
Compound 58	BRD4	Able to overcome drug resistance in cancer treatment.	^115; 141^
Compound 60	BRD4	Highly selective. Reduces tumor growth and lessens cancer symptoms in vivo and in -vitro, with no apparent side effects. It is considered a potential therapeutic in triple-negative breast cancer.	^115; 141^
Naphtho[2,1-b:7,6-b′]difuran-2,8-dicarboxylic acid hydrate (CPA), CPB, AMR	PIM	Lack of cell permeability; hence, it cannot be used clinically.	^146^
8-hydroxy-4-methyl-9-nitrobenzol(g)chrome-2-one (NBC)	PIM	Induces apoptosis.	^138; 147^
1-β-D-2′-deoxyribofuranosyl-4,5,6,7- tetrabromo-1H-benzimidazole (TDB)	PIM	Extremely high selectivity indicates it has clinical potential.	^148^
Compound 66	PIM	Cytotoxic against cancer cells but not healthy cells. Inhibits the proliferation of various cancer cell lines. Reduces the viability of cancer cells more effectively than Silmitasertib. It is membrane-permeable.	^147^
6-(4-Hydroxy-3-methoxybenzylidene)-5-imino-2-(trifluoromethyl)-5H-[1,3,4]thiadiazolo[3,2-a]pyrimidin-7(6H)-one (SRPIN803; CK2 inhibitor XIII).	SRPK1	Inhibits aberrant angiogenesis. Significantly inhibits cell viability in Jurkat cell lines. Prevents the formation of intraocular neovascularization in vivo.	^149^
108600	TNIK, DYRK1	The inhibitory effect on CK2α’ is ten times stronger than on CK2α. Inhibits tumor growth in breast cancer cells and overcomes chemical resistance. *In vitro* and *in vivo* studies suggest it is an optimal inhibitor in clinical settings.	^150^

**Table 3:** CK2 inhibitors with pathway or subunit-specific mechanism of action

Name	Status for Clinical Application	Reference

CIGB-300	Cell-permeable. Inhibits angiogenesis and metastasis. Used in early clinical trials in combination with chemoradiotherapy as a therapeutic against cervical cancer. Administered by injection into the tumor. Targets the phosphoacceptor domain. Releases histamine from the cells, possibly due to higher intracellular calcium levels in the cell.	^42^
4,5,6,7-tetrabromobenzotriazole (TBBt)	Moderately effective as an anti-cancer drug. Induces apoptosis in tumor cells. Inhibits CK2α subunit. They are used in Sepsis-Induced Acute Kidney Injury.	^154; 155^
4,5,6,7-tetrabromobenzimidazole (TBBz)	Able to target specific molecular forms of CK2. It is more effective in inducing apoptosis and necrosis in tumor cells than TBBt. Inhibits CK2α subunit activity. It was tested in Glioblastoma Cell lines.	^154; 156^

## Data Availability

No new data were created or analyzed in this study. Data sharing is not applicable to this article.
